# Supplementary material for: Polymer morphology and interfacial charge transfer dominate over energy-dependent scattering in organic-inorganic thermoelectrics
Source: Nat Commun. 2018 Dec 17;9:5347. doi: 10.1038/s41467-018-07435-z (PMC6297356; doi:10.1038/s41467-018-07435-z)
Supplement: Supplementary file 1 — Supplementary Information [file 41467_2018_7435_MOESM1_ESM.pdf]

Supplementary Information for Polymer morphology and interfacial charge transfer dominates over energy-dependent scattering in organic-inorganic thermoelectrics

Pawan Kumar, Edmond W. Zaia*, Erol Yildirim, DV Maheswar Repaka, Shuo-Wang Yang, Jeffrey J. Urban[#], Kedar Hippalgaonkar[#]*

**equal contribution, [#]corresponding authors*

Supplementary Figures

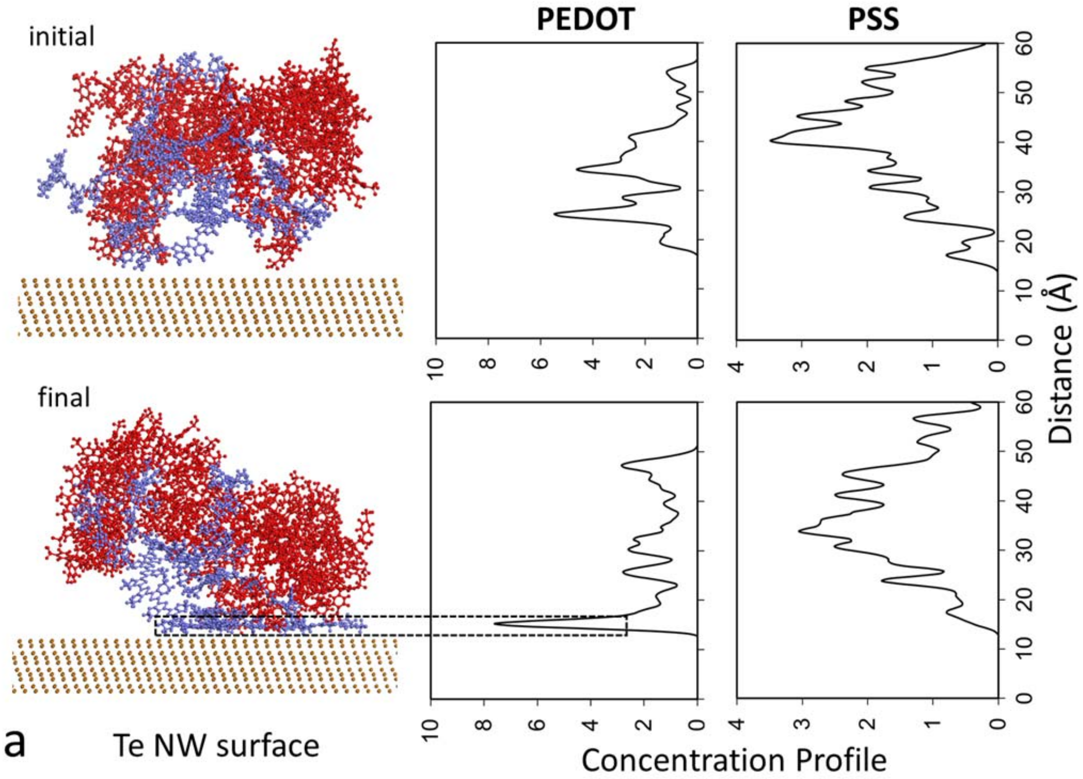

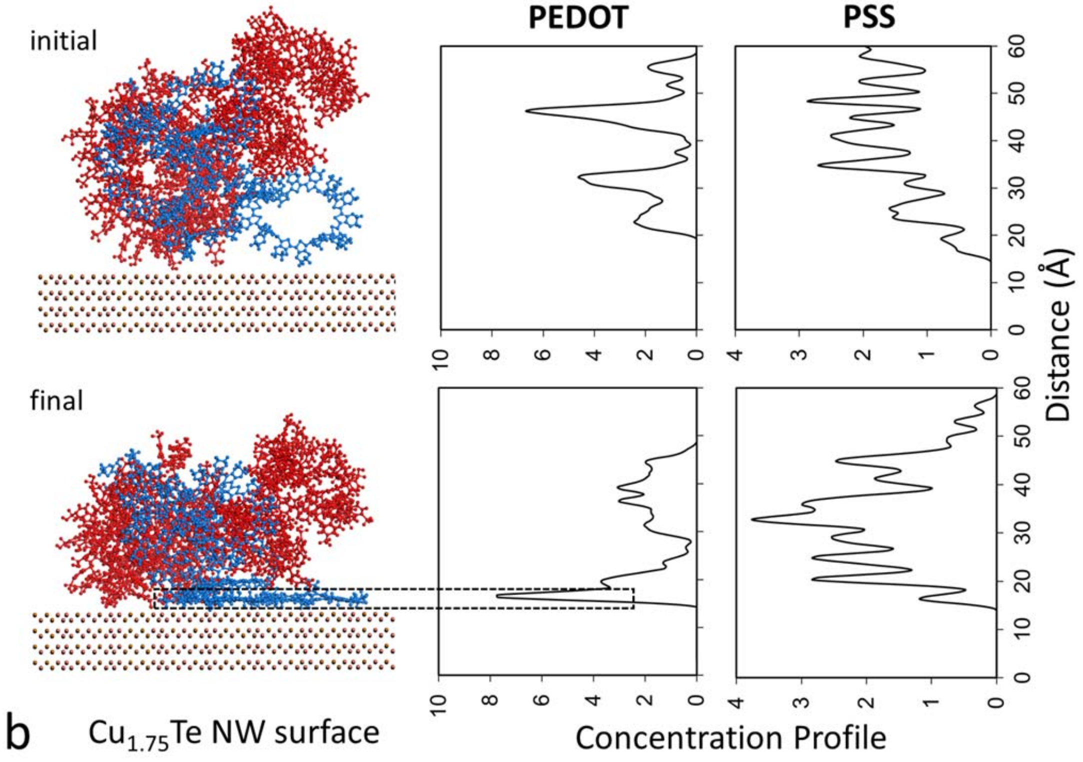

Supplementary Figure 1. MD Simulations of polymer morphology on inorganic surfaces. Initial-final structures and corresponding concentration profiles for MD simulation PEDOT and PSS on **a)** Te and **b)** $Cu_{1.75}Te$ surface. Note that PEDOT and PSS are represented as blue and red chains respectively.

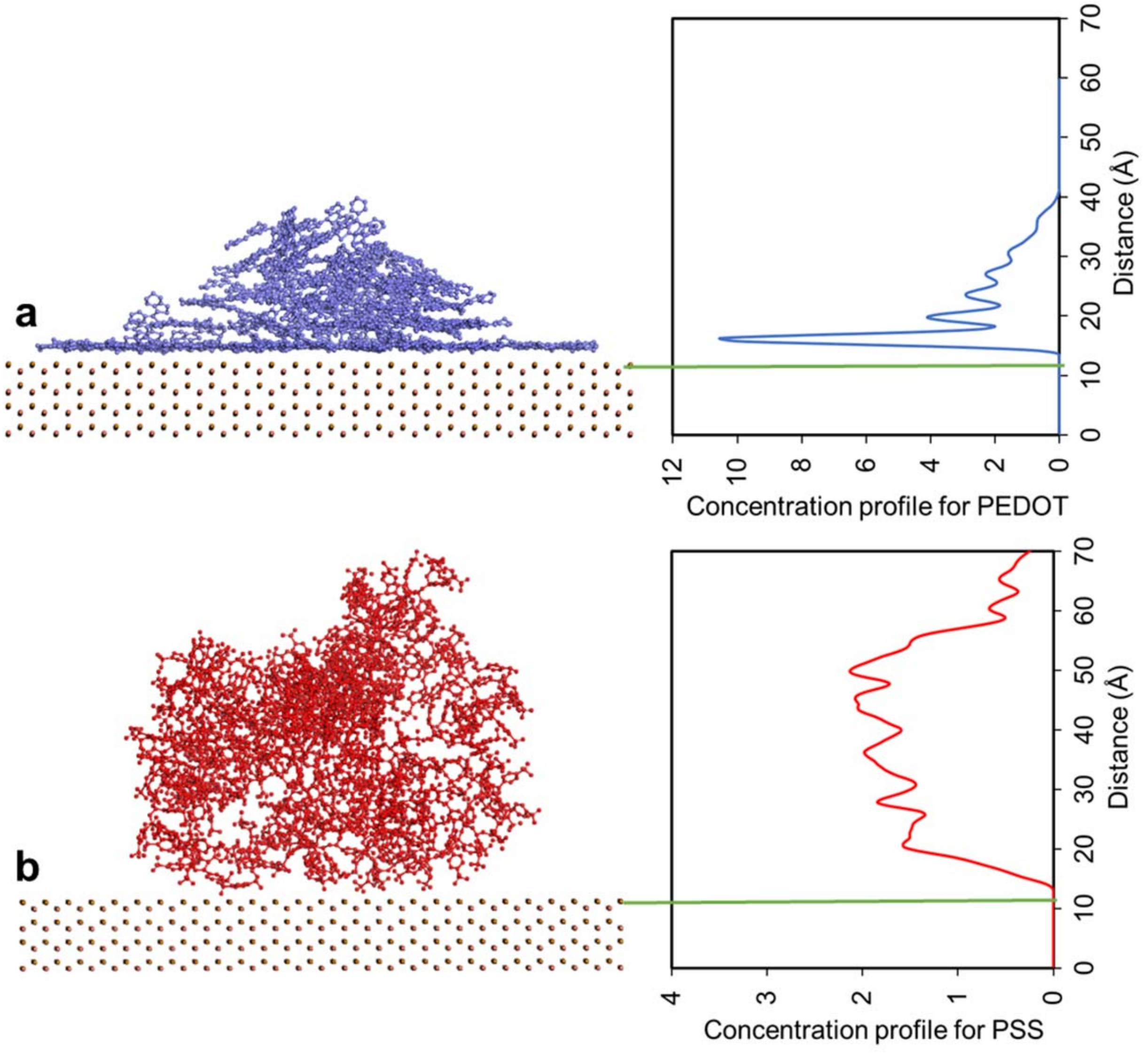

Supplementary Figure 2. MD simulations of pristine polymers on inorganic surfaces. Final structures for MD simulation of a) pristine PEDOT and b) pristine PSS on planar $Cu_{1.75}Te$ surface and concentration profile for these structures.

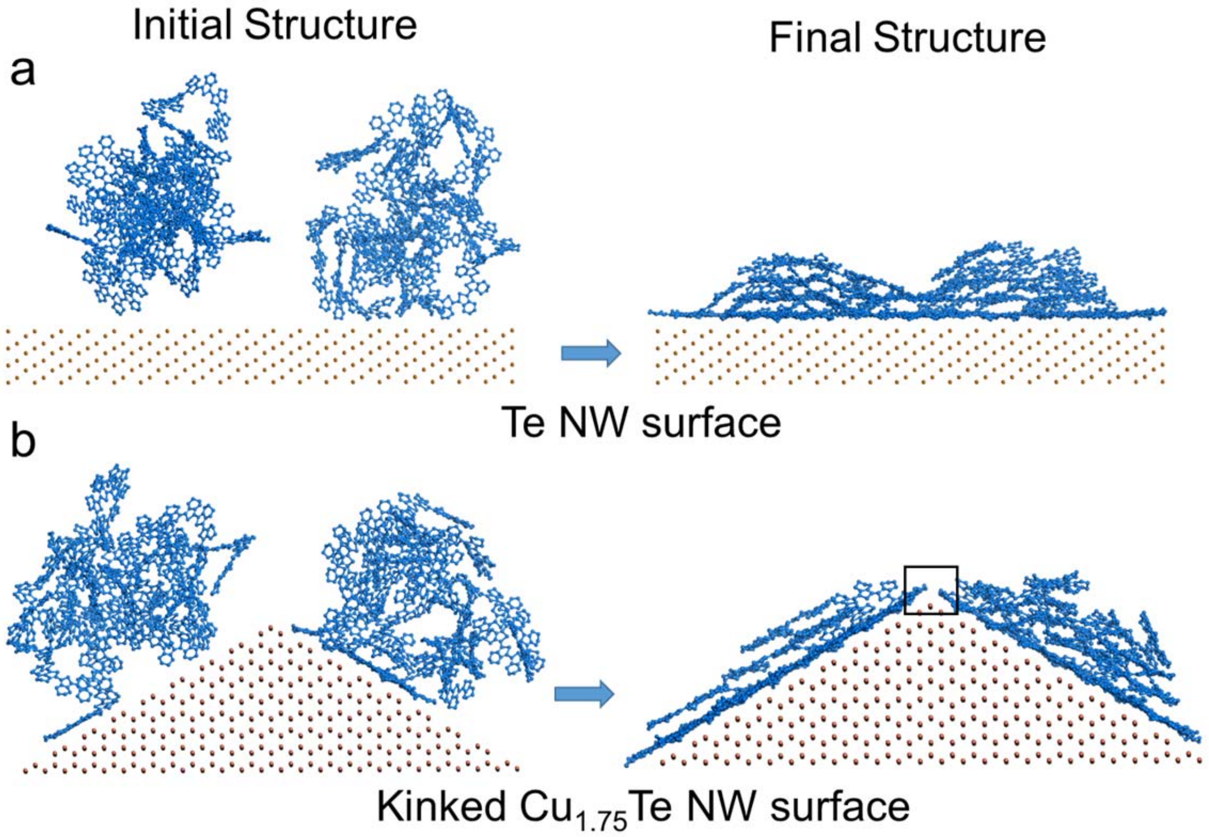

Supplementary Figure 3. MD simulation of PEDOT on flat or kinked surfaces. a) Percolation of two set of $PEDOT_{18}$ chains on Te surface. b) Reduced percolation of PEDOT chains on $Cu_{1.75}Te$ surface. Two sets of ten $EDOT_{18}$ chains were used on each surface.

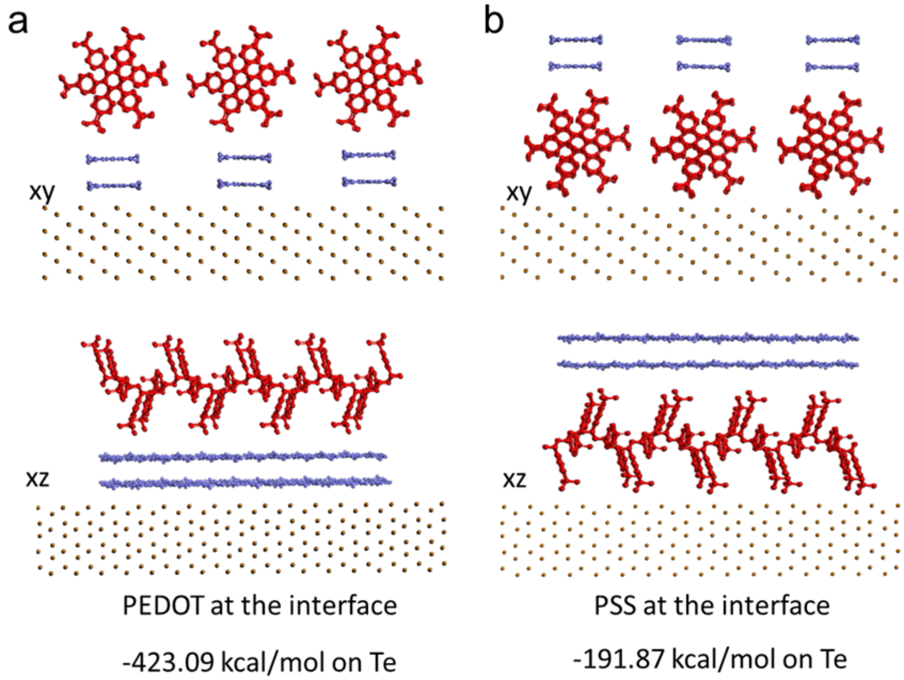

Supplementary Figure 4. Comparison of interaction energies between PEDOT or PSS and the Te surface. Interaction energies are considered for a) six PEDOT and b) three PSS oligomer chains on the Te nanowire interface.

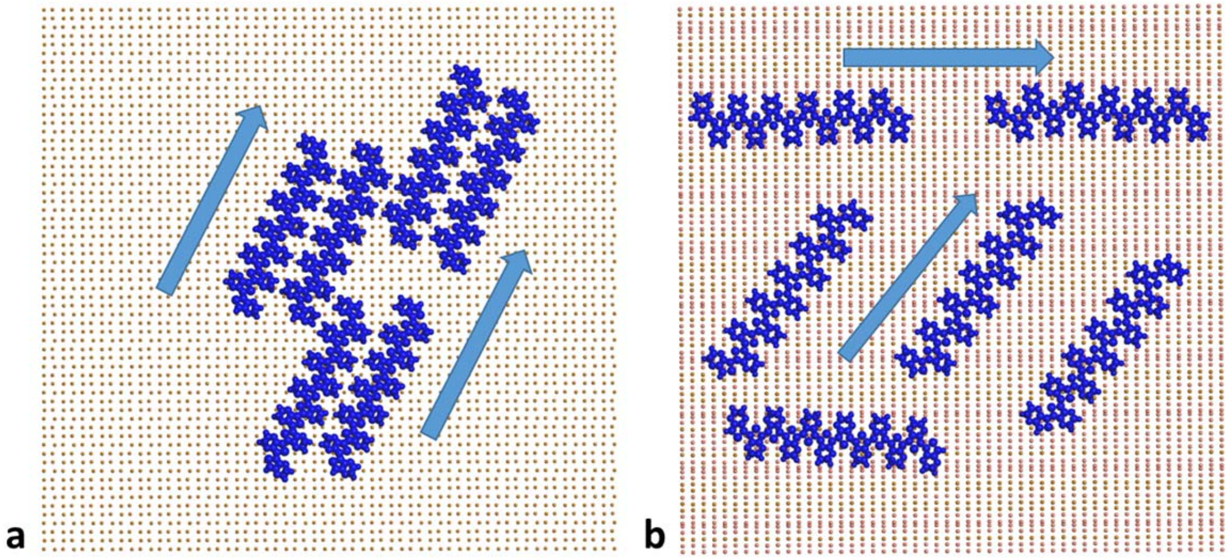

Supplementary Figure 5. MD simulations reveal PEDOT equilibrium structures on the inorganic surface. One of the equilibrium structure from MD simulations demonstrated a) self-assembly and self-alignment of PEDOT chains on Te surface, b) self-alignment of PEDOT chains on $Cu_{1.75}Te$ (no self-assembly) surface.

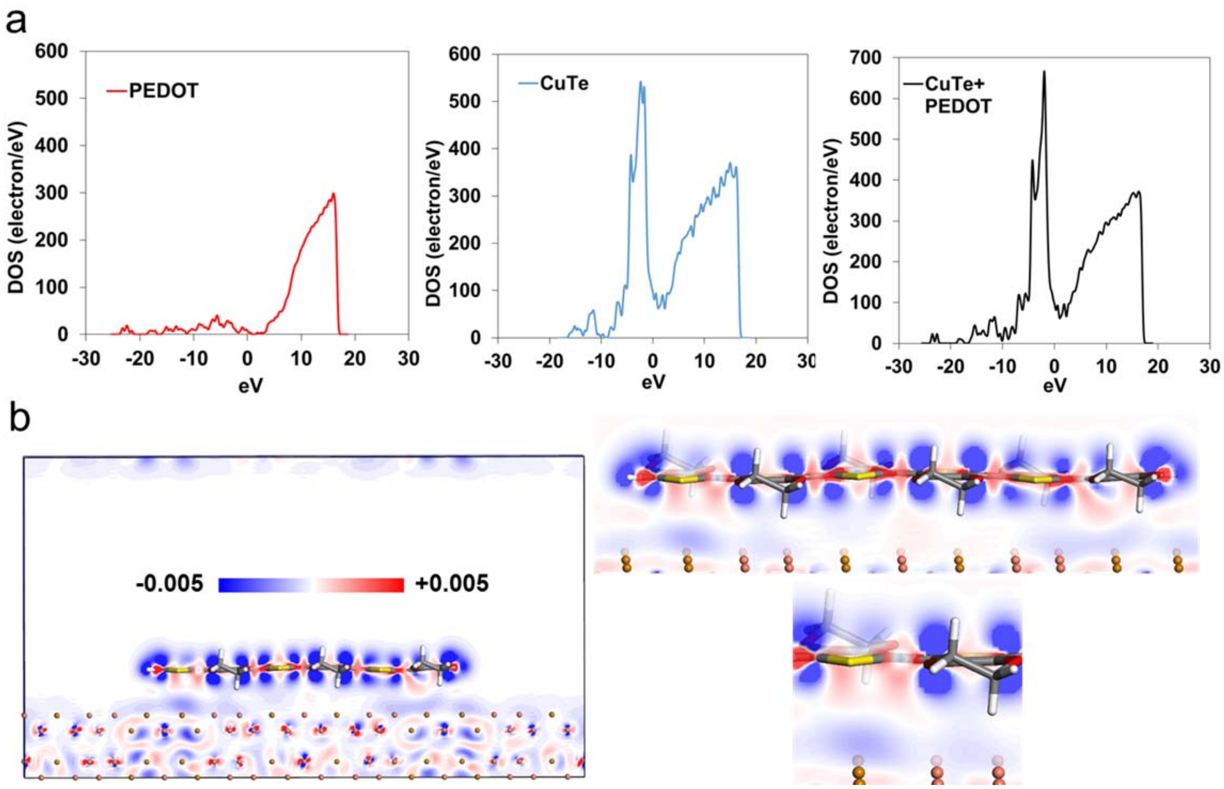

Supplementary Figure 6. Density functional theory calculations for PEDOT on $Cu_{1.75}Te$ surface. A) Total Density of States (DOS) for PEDOT on $Cu_{1.75}Te$ surface, PEDOT and $Cu_{1.75}Te$ surface. Similar with Te-PEDOT interface; density of States (DOS) calculated for (i) PEDOT, (ii) the $Cu1_{.75}Te$ surface, and (iii) the hybrid structure (Figure 2), also depict a trivial change in DOS energies between the individual and hybrid structures. B) Electron density difference for PEDOT hexamer on the $Cu_{1.75}Te$ surface that illustrate electron transfer from $Cu_{1.75}Te$ surface to PEDOT oligomer and intra-chain electron transfer within the PEDOT oligomer.

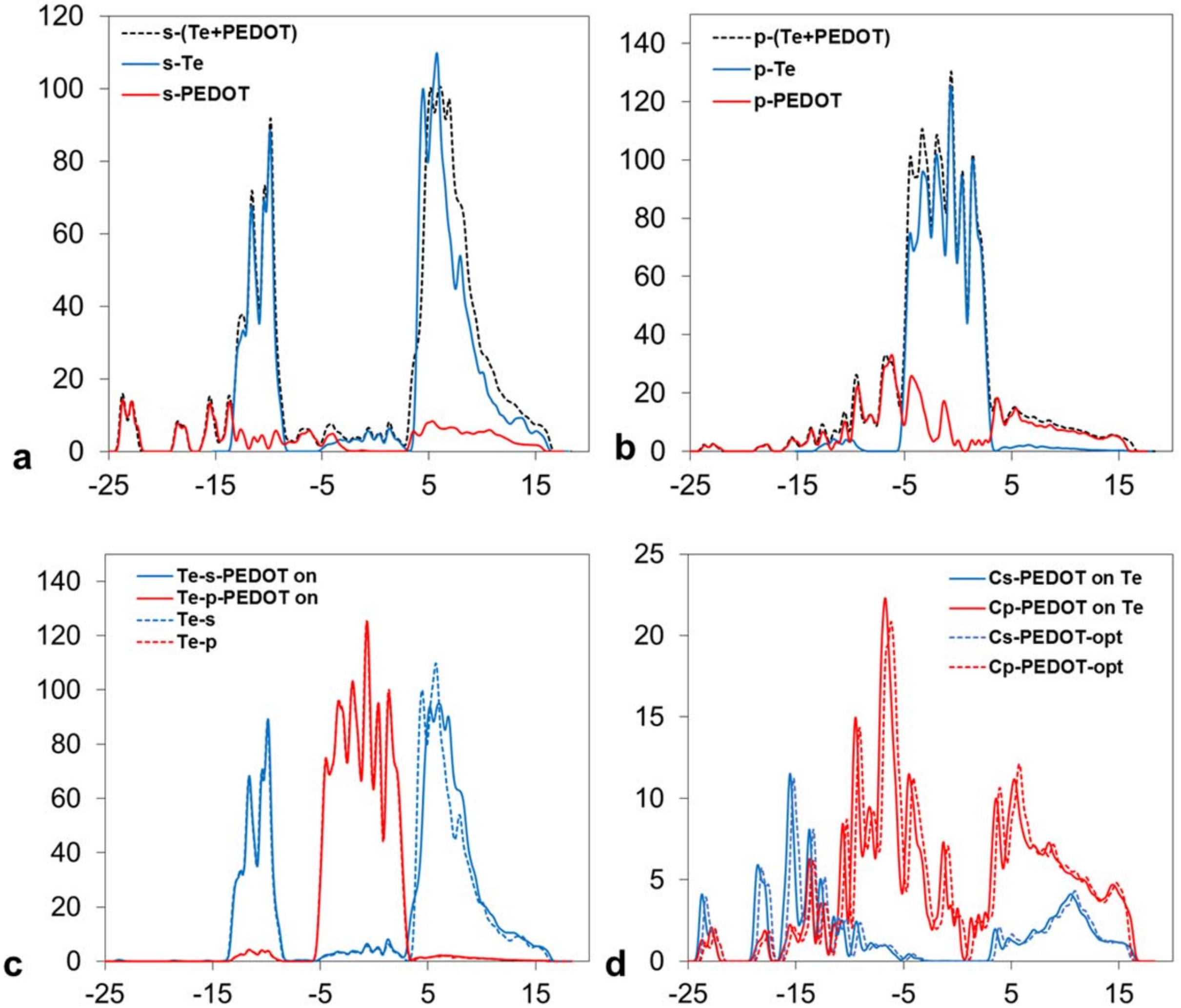

Supplementary Figure 7. Partial density of states are calculated for relevant hybrid systems. Partial density of states for a) s-orbitals of PEDOT-Te NW, Te NW and PEDOT hexamer b) p-orbitals of of PEDOT-Te NW, Te NW and PEDOT hexamer, c) s and p-orbitals of Te NW with and without PEDOT, d) s and p-orbitals of PEDOT with and without Te NW.

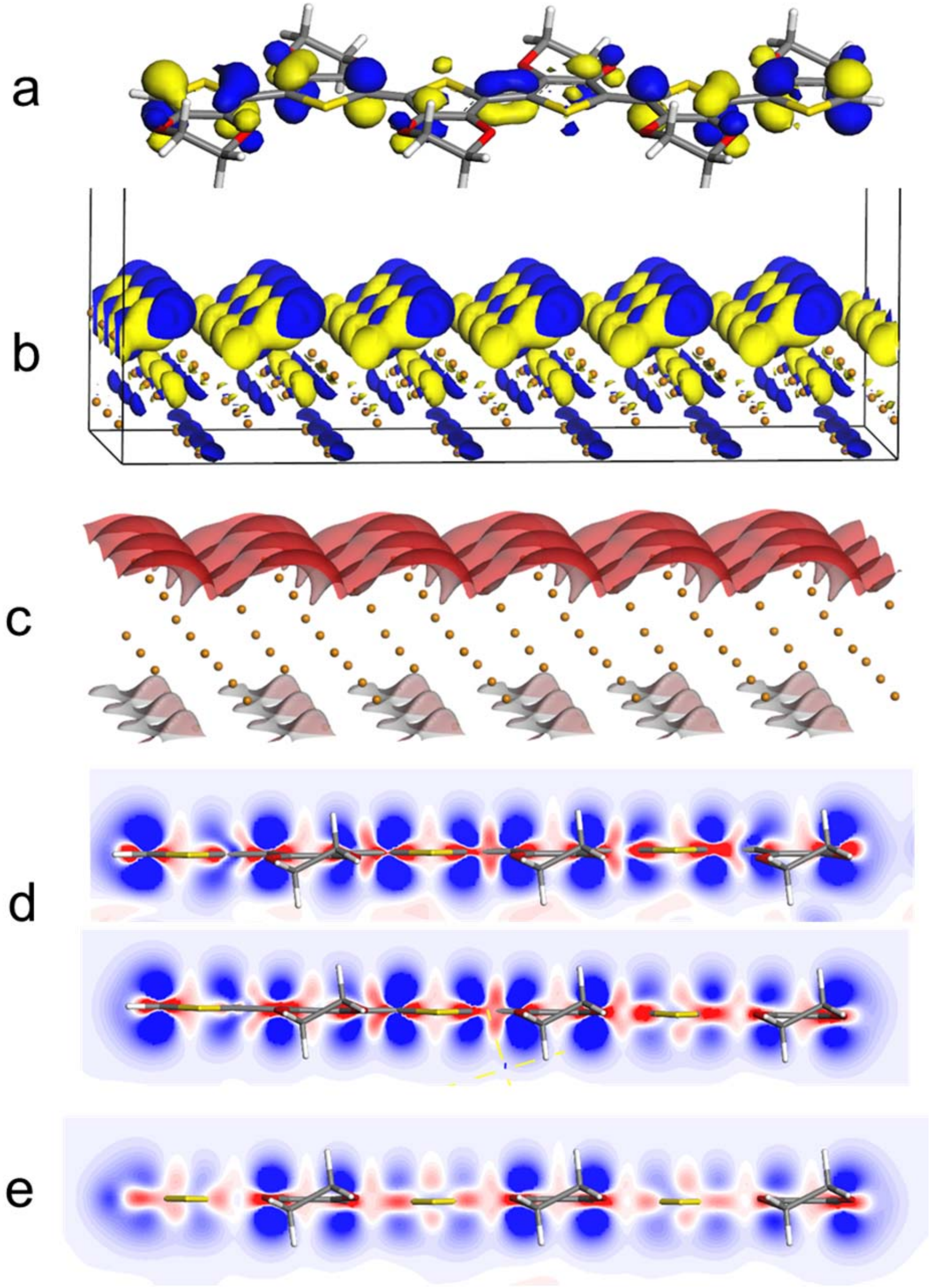

Supplementary Figure 8. Orbital and electrostatics calculations. a) HOMO for $PEDOT^{+2}$ hexamer on Te surface, b) HOMO for Te surface, c) Electrostatic Potential Surface for Te NW surface, d) Electron density difference for $PEDOT^{+2}$ charged hexamer radical for two different direction on the Te nanowire surface e) Neutral PEDOT hexamer show lower charge density difference compared to polaron on Te nanowire surface.

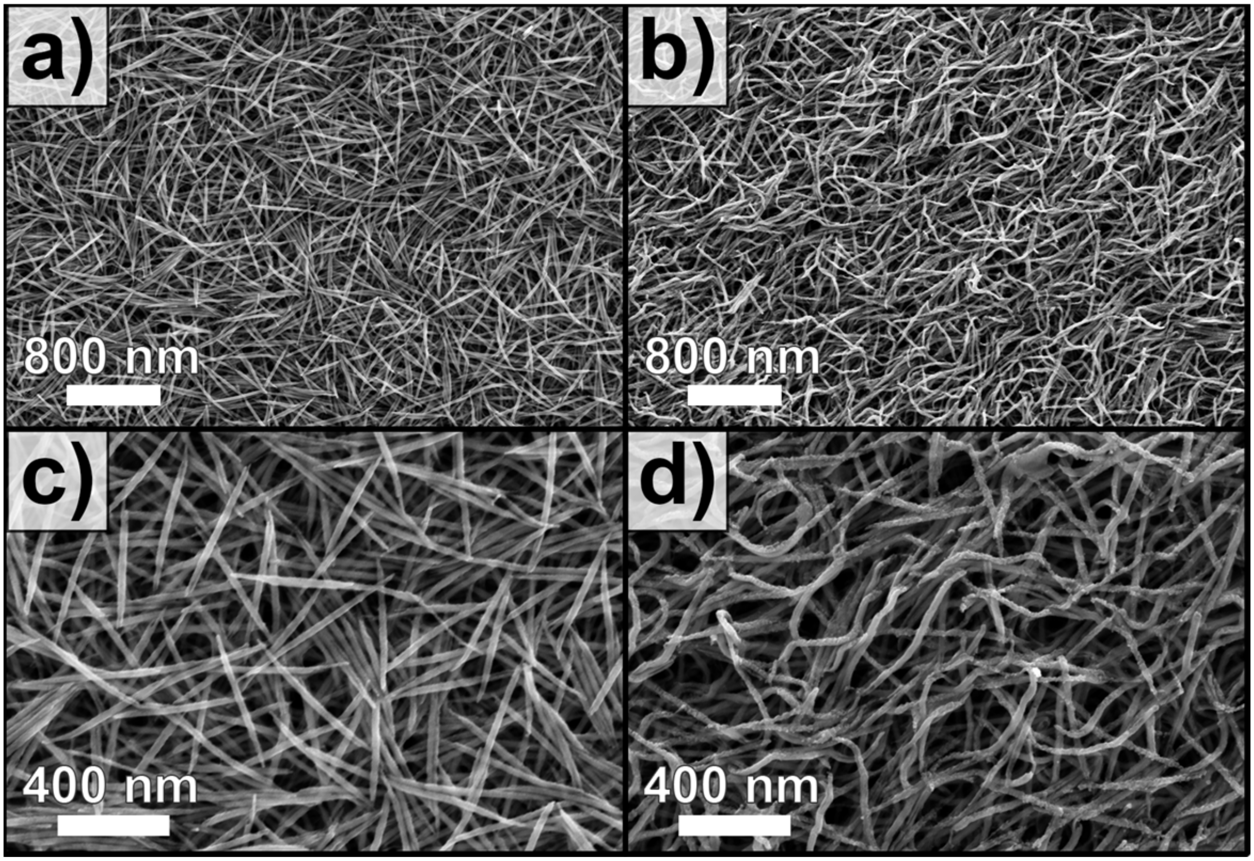

Supplementary Figure 9. Scanning electron microscopy images depict high quality, dense thin films of PEDOT:PSS-Te(Cu_x) NWs. PEDOT:PSS-Te NWs prior to any Cu incorporation show straight, rigid morphology, shown here at (a) 20kx and (c) 50 kx magnification. PEDOT:PSS-Te(Cu_x) with 40% Cu demonstrate 'kinked' morphology indicative of the growth of $Cu_{1.75}Te$ alloy subphases within the NWs, visible at both (b) 20kx and (d) 50kx magnification.

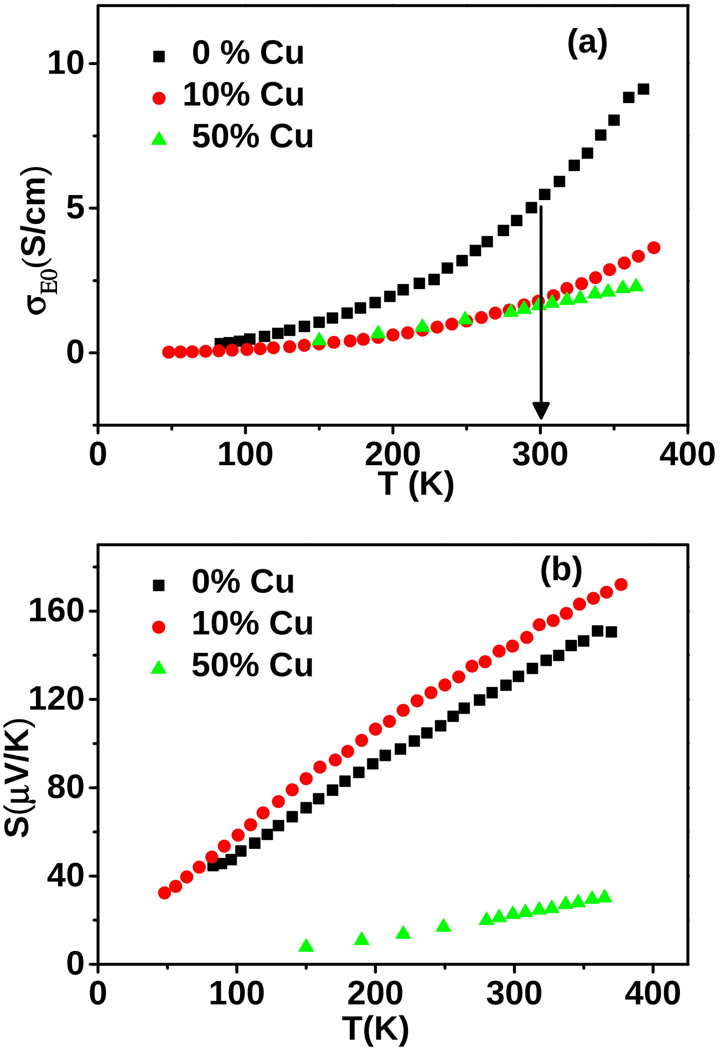

Supplementary Figure 10. Temperature dependent thermoelectric measurements. Temperature dependent (a) conductivity (σ_{E0}) and (b) Seebeck coefficient for PEDOT:PSS-$TeCu_x$ hybrid samples with different Cu loading.

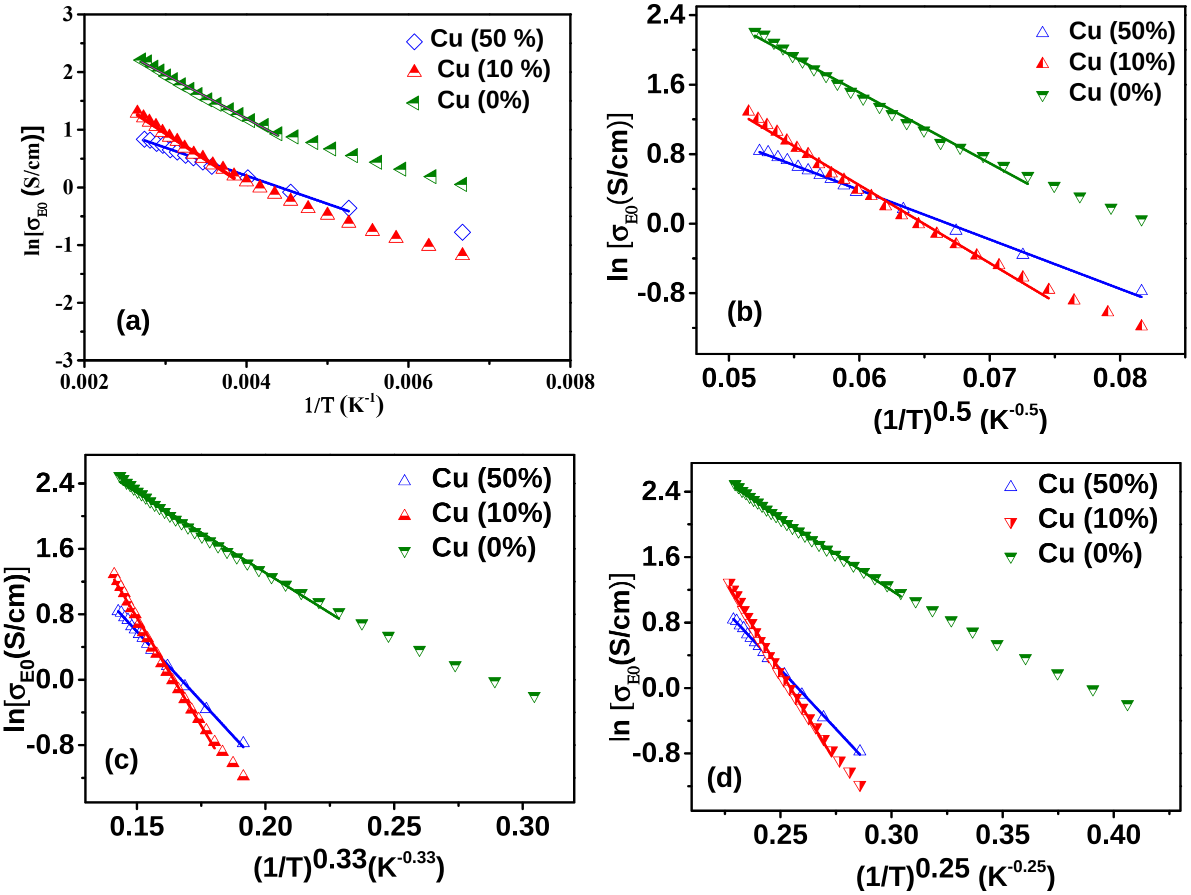

Supplementary Figure 11. Fitting of temperature dependent conductivity measurements. $\mathrm{Ln}(\sigma_{E0})$ as a function of $T^{-\gamma}$ where (a) $\gamma=1$ corresponds to activation energy model. γ is taken as 0.5, 0.33 and 0.25 for 1D, 2D and 3D hopping as shown in (b), (c) and (d). W_{γ} was extracted by taking slope of $\mathrm{Ln}(\sigma_{E0})$ vs $T^{-\gamma}$ curve.

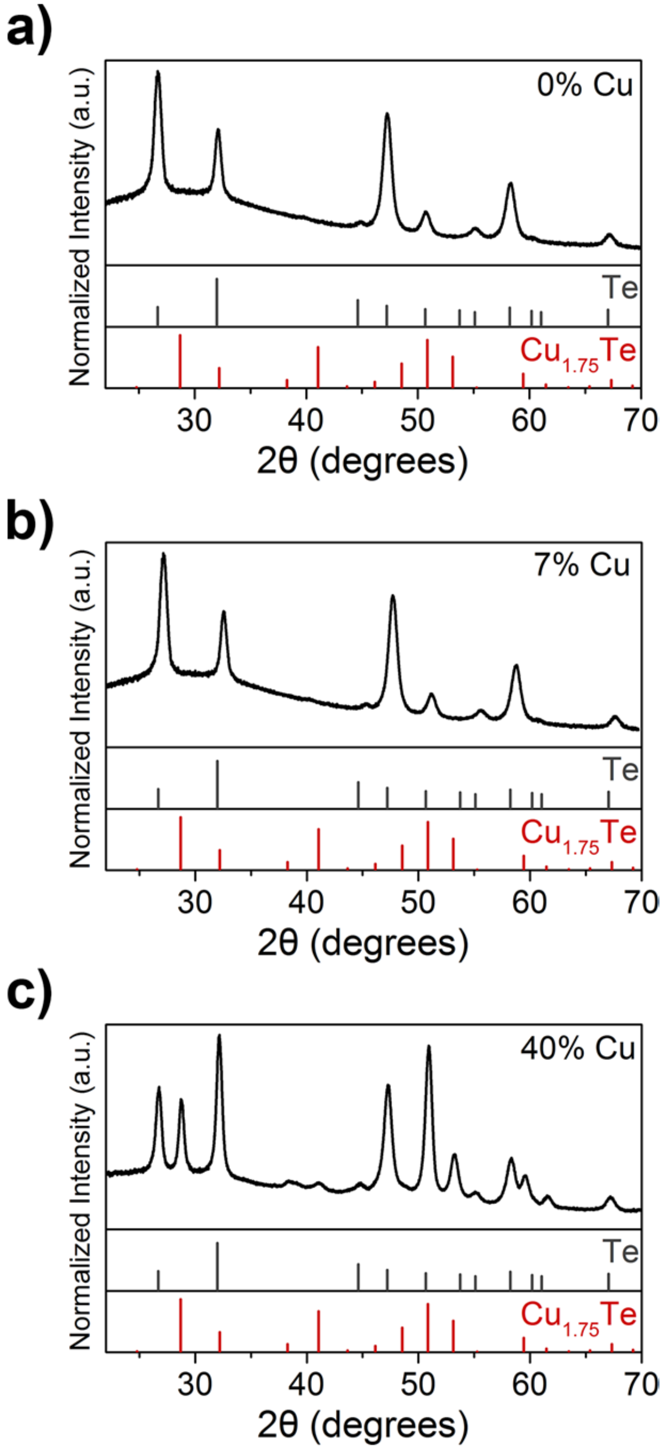

Supplementary Figure 12. XRD spectra of PEDOT:PSS-Te(Cu_x) NWs with different amounts of Cu loading. (a) Prior to any Cu addition, the XRD spectra match reference spectra for Te (ICDD PDF-4 #04-016-1605). (b) and (c) Upon addition of Cu, peaks associated with $Cu_{1.75}Te$ sub-phases (ICDD PDF-4 # 04-007-0008) can be observed. This effect is most pronounced in high Cu loading samples, as seen in panel (c).

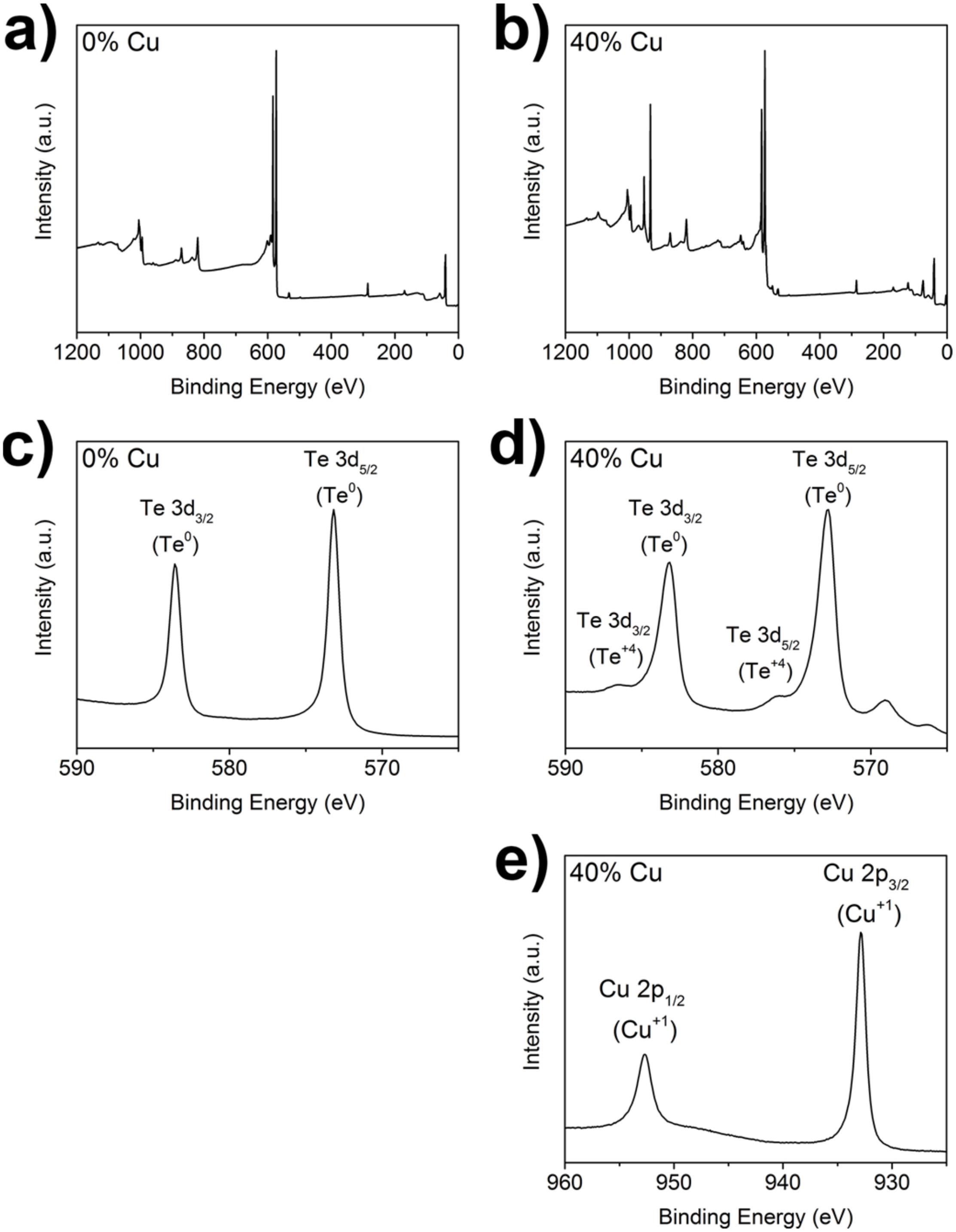

Supplementary Figure 13. XPS was used to characterize the surface composition and valence states of Cu and Te in thin films of PEDOT:PSS-Te(Cu_x) NWs. (a) and (c) In PEDOT:PSS-Te NWs (0% Cu), Te 3d spectra show that the nanowires are composed of fully reduced Te^0 as expected. (b), (d), and (e) PEDOT:PSS-Te(Cu_x) NWs (40% Cu) show weak Te^{+4} satellites corresponding to the growth of minority oxide species such as TeO_2. Additionally, Cu 2p XPS confirms that Cu atoms are primarily in the Cu^{+1} valence state, consistent with the growth of $Cu_{1.75}Te$ sub-phases.

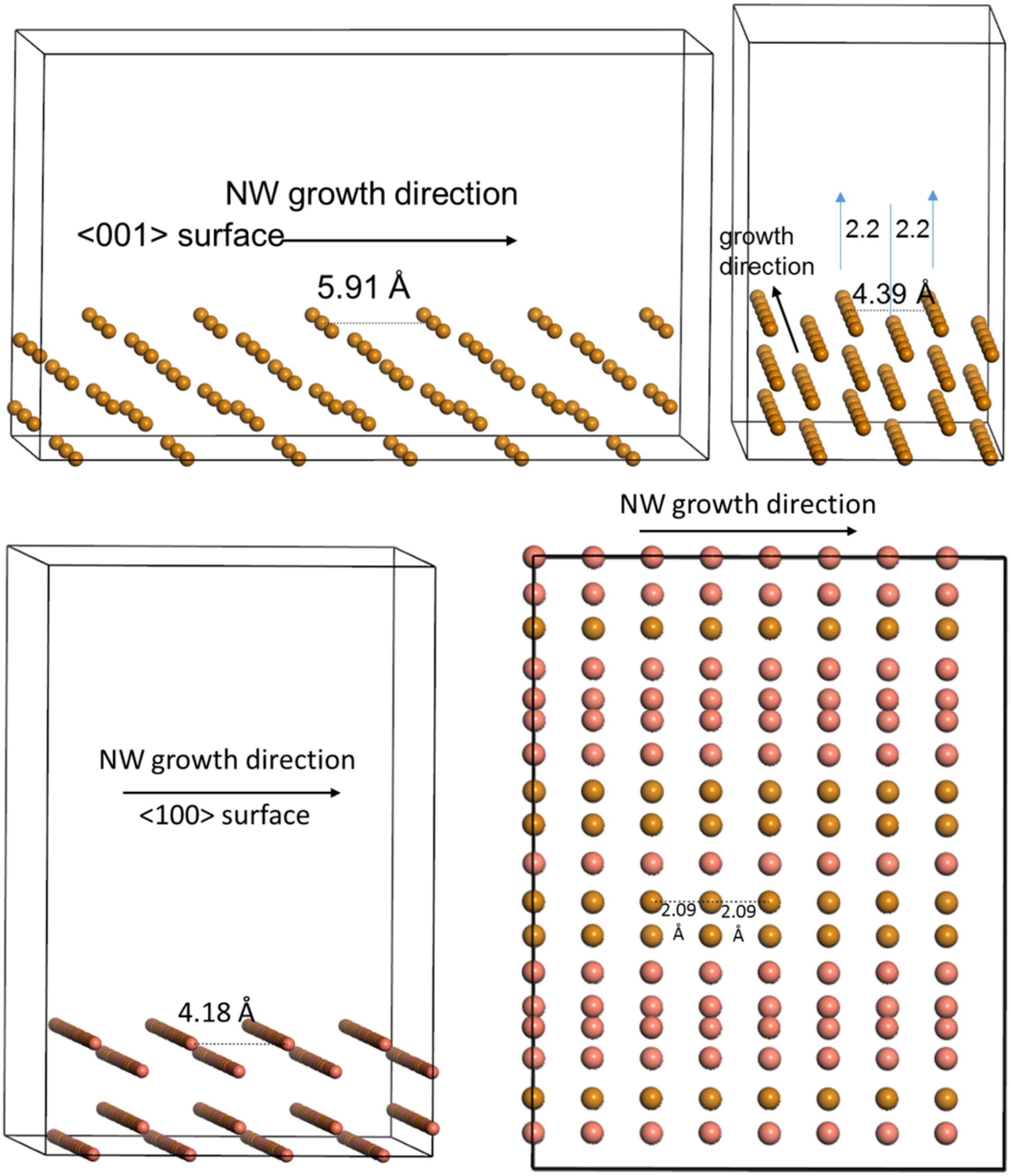

A

B

Supplementary Figure 14. Nanowire surface models. a) Te and b) $Cu_{1.75}Te$ nanowire surfaces models constructed according to experimental results

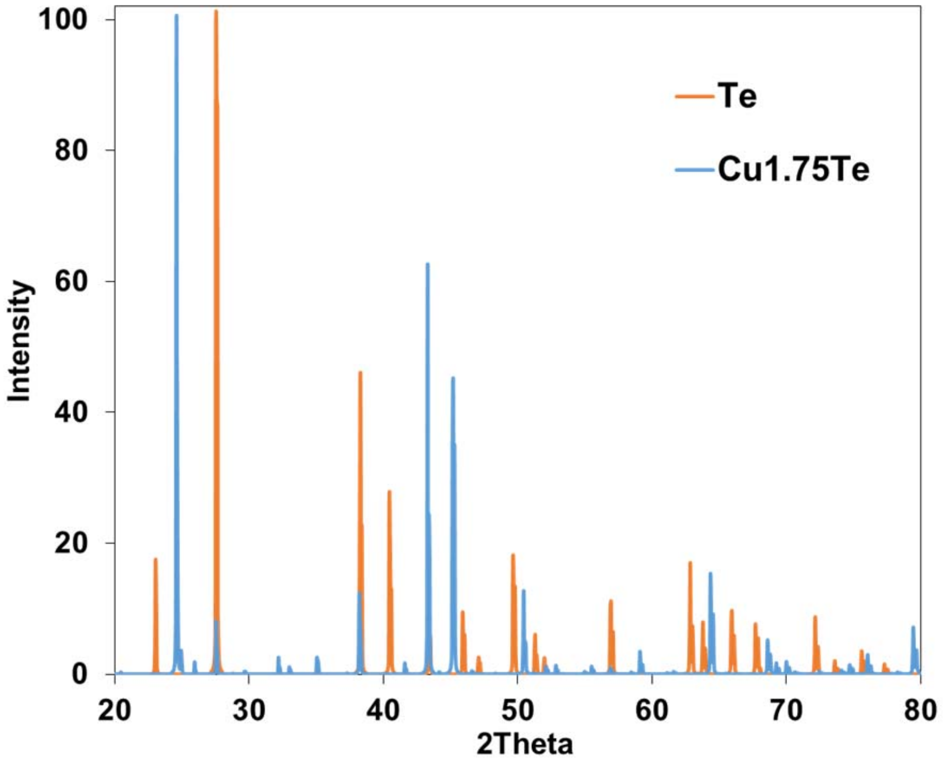

Supplementary Figure 15. Calculated XRD powder diffraction data for Te and $Cu_{1.75}Te$ unit cells. Good agreement is shown with the experimental studies.

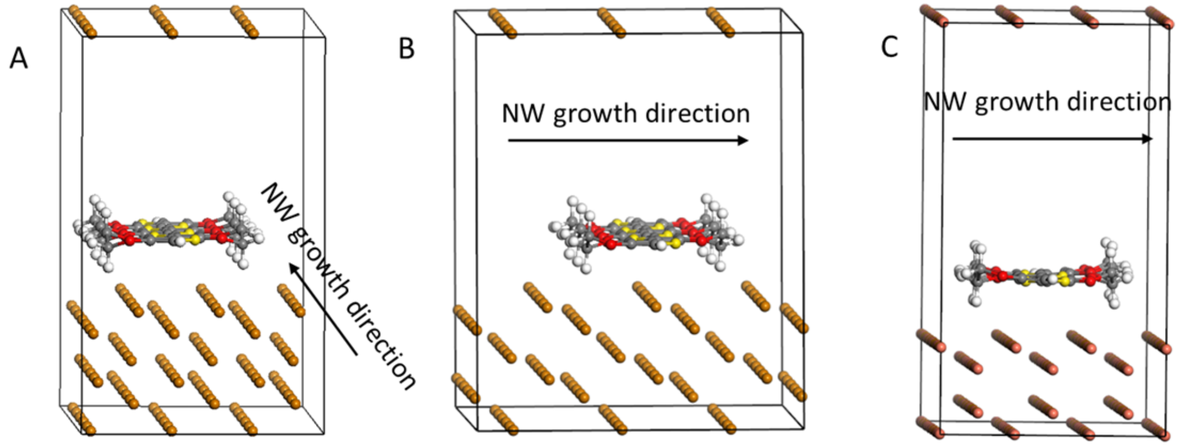

Supplementary Figure 16. Initial structures for DFT calculations of PEDOT hexamer on the surface. Two directions calculated for Te surface **a)** along to the growth direction, **b)** perpendicular to the growth direction, **c)** PEDOT hexamer on the $Cu_{1.75}Te$ surface.

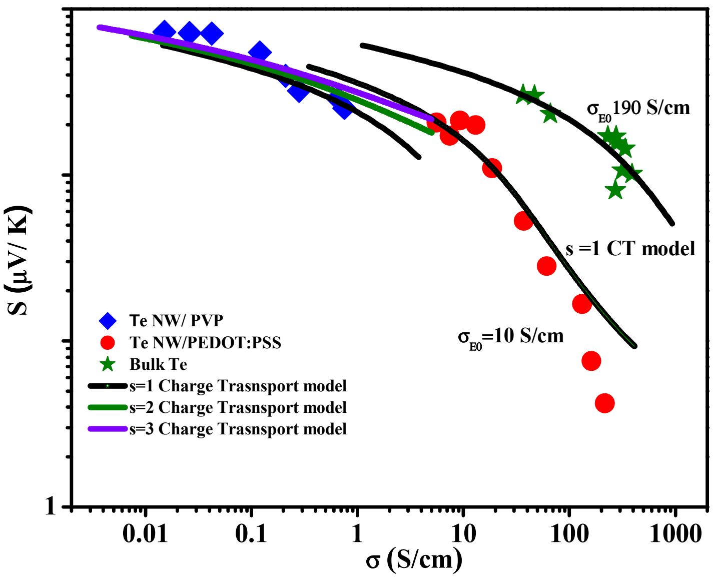

Supplementary Figure 17. Experimental data of Seebeck (S) vs conductiviy (σ) for Te(Cu_x) nanowire systems. Te(Cu_x) nanowires embedded in insulating polymer matrix (closed square), PEDOT:PSS-Te(Cu_x) NW hybrid system (closed circles) and bulk Te (closed star) modelled with s=1(solid lines). It is seen Seebeck vs conductivity data of PEDOT-based hybrid system and bulk Te lies on s=1 curve with different σ_{E_0} transport coefficient values. In case of Te/CuTe nanowire embedded in insulating polymer matrix, the Seebeck vs conductivity data lies on all possible s values (s=1, 2, and 3) and it is difficult to distinguish which 's' is true for the system.

Supplementary Tables

Supplementary Table 1. Charge transfer and de-doping effect for the pristine and doped PEDOT chains on Te and $Cu_{1.75}Te$ surfaces for the geometry optimized structures.

	**electron/monomer (Mulliken)**	**De-doping effect* (electron/cm^3)**
Neutral $PEDOT_6$ on Te	-0.078 for 3.6 Å<br>(-0.016 for 8 Å)<br>(none over 15 Å)	-6.19×10^{20} for 3.6 Å<br>(-1.27×10^{20} for 8 Å)<br>(none over 15 Å)
$PEDOT_6^{+2}$ on Te	-0.186	-1.56×10^{21}
Neutral $PEDOT_6$ on $Cu_{1.75}Te$	-0.144	-1.14×10^{21}
$PEDOT_6^{+2}$ on $Cu_{1.75}Te$	-0.239	-2.05×10^{21}

*PEDOT monomer volume 1.26×10^{-22} cm^3

Supplementary Table 2. 1D, 2D and 3D modeling on the Kang-Snyder model for different levels of Cu loading in the hybrid system.

**γ**	**W_g (eV) for 0% Cu loading**	**W_g (eV) for 10% Cu loading**	**W_g (eV) for 50% Cu loading**
**1**	**0.065**	**.079**	**0.041**
**0.5**	**0.57**	**0.69**	**0.28**
**0.33**	**10.5**	**12.6**	**3.4**
0.25	192	343	61.6

Supplementary Notes

Supplementary Note 1. Molecular dynamics simulations of the interaction between PEDOT and PSS on inorganic surfaces.

The self-alignment and self-organization of PEDOT chains at interface was determined for both Te and $Cu_{1.75}Te$ surfaces by calculating the concentration profiles of sulfur atoms in the PEDOT and PSS chains. PEDOT and PSS chains are represented by blue and red in color, respectively. This phenomenon can be seen more clearly by comparing with the initial structures and concentration profiles before and after simulated annealing (Supplementary Figure 1). We did not observe any significant phase separation between PEDOT and PSS chains nor diffusion of PEDOT chains for the simulations of mixed PEDOT:PSS on Te and $Cu_{1.75}Te$ surfaces, unlike strong self-organization and alignment seen in the pristine PEDOT (Supplementary Figure 2a, Supplementary Movies 3-4). In fact, simulations of pristine amorphous PSS on $Cu_{1.75}Te$ NW show that it remains unperturbed on the NW surface (Supplementary Figure 2b, Supplementary movie 5). We attribute these phenomena to a stronger electrostatic attraction between PEDOT and PSS in their core-shell morphology as compared to their isolated interactions with the inorganic phase.[1,2] Note that PEDOT and PSS are represented as blue and red chains respectively throughout the Supplementary Figures.

Further, interaction energies are calculated for six $EDOT_{12}$ and three SS_{24} oligomers with two different configurations where surface is PEDOT rich and PSS rich, respectively (Supplementary Figure 4). Large differences in the interaction energies showed that concentration of PEDOT chains is higher at the interface compared to the PEDOT:PSS bulk, which has higher PSS concentration. Combining this result with those in Figure 1 and Supplementary Figure 2, the Te nanowire-PEDOT:PSS interface is not only PEDOT rich, but also highly self-organized. The self-aligned PEDOT chains present at the interface are likely a key component to the enhanced TE performance of PEDOT:PSS hybrid materials. Interaction energies calculated for six EDOT12 and three SS24 oligomers with two different configurations where surface is PEDOT rich and PSS rich, respectively. High difference in the interaction energies showed that concentration of PEDOT chains are higher at the interface compared to PEDOT:PSS bulk which has higher PSS concentration. Taking into consideration with results from Figure 1 and Figure S2, nanowire-PEDOT:PSS interface is not only PEDOT rich, but also

highly self organized and self aligned PEDOT chains present at the interface which is one the origin of the enhanced TE performance of PEDOT:PSS hydrid materials.

In light of the strong indication suggesting that PEDOT self-assembles at the nanowire surface, it is important to identify if there is any directional preference for these chains at the organic-inorganic interface. For this goal, simulated annealing method (details in Supplementary Methods) is applied to six pristine PEDOT oligomers randomly dispersed as monolayers onto the Te and $Cu_{1.75}Te$ surface to determine self-organization and directional preference for these chains. Final structures for self-assembly and self-alignment of PEDOT chains on Te surface and self-alignment of PEDOT chains on $Cu_{1.75}Te$ surface are given in Supplementary Figure 5. Self-assembly of PEDOT chains were not observed on $Cu_{1.75}Te$ surface due to stronger chain interaction with the surface.

Supplementary Note 2. Density functional theory calculations of charge redistribution in PEDOT at the organic-inorganic interface

We calculated charge transfer quantitatively based on the Mulliken charges for the PEDOT hexamer on the Te and $Cu_{1.75}Te$ surface. Charge transfers were calculated for both neutral PEDOT and charged bipolaron state of PEDOT hexamer for the geometry optimized structure on the surface. Same level of first principle DFT calculations were performed with previous calculations. For the bipolaron case, we added +1 charge for every three monomer as predicted experimentally.[3] Charge transfers are calculated per monomer and minus sign represents electron transfer from inorganic surface to PEDOT chains.

Similar with Te surface, DOS calculations allude to physisorption of PEDOT on the NW surface and the charge density differences indicate intra-chain electron transfer within the PEDOT oligomer due to a similar pillow effect on the $Cu_{1.75}Te$ surface (Supplementary Figure 6a-b). Further insight into this observed pillow effect at the PEDOT-NW interface is provided by calculation of the highest occupied molecular orbitals (HOMOs) and electrostatic potentials of both PEDOT and Te at the surface (Supplementary Figure 8a-c). As a result, the C-C bond distance between $(EDOT_6)^{+2}$ monomers is reduced from 1.42 to 1.40 Å when in proximity to the Te surface. As discussed in the main text, such bond shortening is characteristic of a shift from benzoid to quinoid conformation in agreement with reports on the narrowing of C-C stretching bands in studies of PEDOT on graphene oxide surfaces.[4] This may be a factor in the enhanced

conductivity observed in the PEDOT:PSS-Te NW system, as one can expect more linear and extended PEDOT chains (higher conductivity domains) in the quinoid conformation on the Te NW surface compared to the benzoid phase.

Supplementary Note 3. Temperature dependent thermoelectric measurements

Supplementary Figure 10(a) shows temperature-dependent and energy independent conductivity, σ_{E_0}(T) as a function of temperature. At 300 K, as Cu content increases from 0% to 10 % in PEDOT:PSS-TeCu$_x$ system, the conductivity deceases drastically as shown by the arrow. With further loading of Cu, the conductivity does not change much. Conductivity of 0% and 10 % Cu samples decreases rapidly with temperature with similar curvature and tends towards to zero as temperature reaches 0K. On the other hand, the conductivity of 50% Cu loaded sample shows almost a linear change with temperature. Supplementary Figure 10(b) shows temperature dependent Seebeck coefficient of all 3 samples. The room temperature value of Seebeck coefficient was found to be 129 μV/K for 0%Cu loading sample. As Cu loading increases to 10%, the Seebeck coefficient enhances and reaches 147 μV/K. For 50 % Cu loading, the Seebeck coefficient drops rapidly and reaches 23 μV/K. The Seebeck coefficient of all 3 samples decreases smoothly with temperature. There are two possibilities for this effect, both of which have literature precedents. First, it has been shown that $Cu_{1.75}Te$ nanowires can demonstrate extremely high conductivities even in the absence of PEDOT:PSS or another conducting organic phase.[5] Thus, it is likely that at high copper loading the inorganic contribution to transport significantly changes. There may then be a transition from PEDOT-dominated transport to $Cu_{1.75}Te$ dominated transport which would cause a deviation from the model predictions. However, the increased conductivity is seen to result in a stronger reduction in the Seebeck, therefore moving away from optimally doped high powerfactor. Additionally, it is known that high levels of molecular dopants can significantly alter the morphology of conducting polymeric domains. That is to say, in the 10-50% copper region, the transport trends observed are the result of purely doping effects, tracked with η. Above 50% copper, the morphology of the PEDOT domains is sufficiently compromised such that both η and $\sigma_{E_0}(T)$ are affected.

Supplementary Figure 11 shows temperature-dependent and energy independent conductivity, σ_{E_0}(T) as a function of $T^{-\gamma}$. Different values of γ (1, 1/2, 1/3, and 1/4) are taken to

analyse the data as shown in (a), (b), (c) and (d), respectively. The value of γ= 1/2, 1/3, 1/4 correspond to 1D, 2D and 3D hopping. The W_γ was extracted using Supplementary Equation 1:

$$\sigma_{E0} \propto \exp -(\frac{W_\gamma}{k_B T})^\gamma \quad (1).$$

The W_γ value is shown in Supplementary Table 2 for different values of γ. For γ =1, while the W_γ value for 0% and 50% Cu loading samples are close (within 25% of each other), the value decreases drastically for the 50% Cu loaded sample. As discussed by Kang and Snyder, for polyacetylene samples (s=3, γ =1/2) with different conductivities, the value of W_γ was found to be constant. If we observe the temperature dependent curvature for these different polyacetylene samples, the curvature is similar.[6] But for higher doping as shown in Y.W. Park et. al.[7], the curvature of conductivity changes and the value of W_γ will not be the same as the universal value. We observe a similar change in the curvature of conductivity against temperature when the Cu loading changes from 10 % to 50% as shown in Supplementary Figure 11(a) and therefore, the value of W_γ decreases drastically for the 50 % Cu loading sample.

Supplementary Note 4. Doping and de-doping mechanism

In order to strengthen our argument that there is no *additional* energy-dependent filtering, we provide extensive theoretical evidence to support our understanding of the mechanisms behind the non-monotonic trends observed in the thermoelectric properties of the PEDOT:PSS-Te/CuTe system. We specifically looked for charge transfer in new DFT simulations and studied their effects on doping/de-doping of the hybrid material. There are three effects that control charge transport in this hybrid system:

First, templating of the conducting PEDOT due to the inorganic (Te/CuTe) nanowire surface, (Table 2, column 2) is defined as self-alignment of PEDOT chains on Te and CuTe atomic surfaces as shown in Fig. S1. Here, the carrier mobility is enhanced and therefore the conductivity increases. There is no change in carrier concentration.

Second, de-doping due to charge transfer at this organic- inorganic interface. We have performed extensive DFT simulations of the inorganic-organic interface to demonstrate that there is indeed charge transfer from the inorganic Te/CuTe to the PEDOT layer as shown by the blue color in Fig. 2 in the revised manuscript. The electron transfer from Te/CuTe to PEDOT can be understood as de-doping of PEDOT (less holes within PEDOT chains, Table 2, column 2).

Third, as described in the original manuscript, Cu+ ion loading results in additional charge carriers being introduced into the conducting PEDOT chains, accompanied with a reduction in the templating effect (Table 2, column 3).

The non-monotonic thermoelectric trends are explained by the interaction of these multiple effects. Upon addition of Cu, the curved $Cu_{1.75}Te$ phase within the Te nanowire expands and weakens the templating and de-doping effects. This would cause a decrease in conductivity and a small increase in Seebeck coefficient compare to pure PEDOT/Te, the effect of which is strongest in the low Cu loading regime. At the same time, not all Cu ions interact with the Te nanowire; instead, some of them dope the PEDOT chains. This doping effect is understood to cause an increase in the conductivity and decrease in the Seebeck coefficient with increasing Cu content, especially apparent in the high Cu loading regime. These conclusions are supported theoretically via MD simulations and DFT calculations.

To understand the thermoelectric properties of PEDOT:PSS-Te, it is important to consider the first two effects listed above. MD simulations show that the PEDOT aligns on the Te surface (i.e. a strong templating is observed), which enhances the mobility of the carriers in the PEDOT chains. On the other hand, due to interfacial change transfer, de-doping occurs giving rise to a large Seebeck coefficient (note that in the Kang-Snyder model, the Seebeck does not depend upon the σ_{E_0} value and instead only on the carrier concentration via the reduced chemical potential, η). The enhancement in mobility compensates for the reduction in carrier concentration that occurs in PEDOT chains relative to the pristine polymer, and therefore both the Seebeck coefficient and electrical conductivity are enhanced. In order to understand this, consider that in order for the Seebeck to be enhanced from ~10 μV/K (pristine polymer) to ~200 μV/K (PEDOT:PSS-Te composite), a carrier concentration decrease of one order of magnitude is required – on the other hand the mobility is expected to increase by two orders of magnitude.

To elucidate the mechanism behind the non-monotonic trend in thermoelectric properties in the full PEDOT:PSS-Te/CuTe system, a combination of all three effects described above is required. First, DFT simulations show that the charge transfer between PEDOT and flat $Cu_{1.75}Te$ surface is stronger than that for PEDOT and Te, resulting in a stronger de-doping effect (reduction in carrier concentration) upon addition of Cu to the system, especially in the low Cu loading regime but, due to curved nature of $Cu_{1.75}Te$, the de-doping effect on PEDOT/ $Cu_{1.75}Te$ surface is not as effective as PEDOT/Te and therefore, Seebeck shows only a small

enhancement (200 to 213 μV/K). Second, as discussed above, the growth of curved $Cu_{1.75}Te$ surface within the Te nanowire weakens the templating, thus decreasing the carrier mobility in the organic phase. Third, Cu loading also dopes the PEDOT chains via ions present in the polymer phase. In the low Cu loading regime, Cu doping (effect 3) is inefficient compared to the charge transfer between PEDOT-Te/$Cu_{1.75}Te$ (effect 2) and therefore, the Seebeck is enhanced. In the case of conductivity, both de-doping (carrier reduction) and weak templating (mobility reduction) will dominate, which is why a reduction in conductivity is observed in this range. As the Cu loading is increased, the curved $Cu_{1.75}Te$ surface with Te nanowire expands, burying the effects of templating and charge transfer induced de-doping, and instead, the doping of PEDOT with Cu (effect 3) will dominate as is confirmed observed experimentally. Combined with the results of fitting using the Kang-Snyder model, our extensive theoretical analysis strongly suggests that this intricate interplay of templating, Cu doping and charge transfer at the interfacial polymer state is responsible for the observed thermoelectric properties, and not a change in the energy-dependence of scattering as was originally proposed.

Supplementary Methods

Materials Synthesis

Syntheses of PEDOT:PSS-Te NWs and PEDOT:PSS-Te(Cu_x) heterostructures were performed following previously reported methods.[8,9] L-ascorbic acid ($C_6H_8O_6$), sodium tellurite (Na_2TeO_3) and copper(II) nitrate hemipentahydrate ($Cu(NO_3)_2$ 2.5H_2O) were purchased from Sigma Aldrich. Acetone (J.T.Baker®), dichloromethane (CH_2Cl_2 – BDH®), isopropyl alcohol (C_3H_8O – ACS Grade), and nitric acid (HNO_3 – BDH®) were purchased from VWR International. These chemicals were used as delivered without further purification. Poly (3,4-ethylenedioxythiophene):poly(styrene sulfonate) (PEDOT:PSS) was purchased from Heraeus as Clevios PH1000. PEDOT:PSS was sonicated and filtered through a 0.45 μm PVDF filter before use in Te NW synthesis.

Thin films (typically 3-5 μm in thickness) were prepared by drop casting on square glass substrates (9.5mm x 9.5mm - Thin Film Devices). Drop casting was performed by placing glass substrates on a large aluminum block (thermal reservoir) at 85°C, then depositing 75 μL of NW solution (typically ~20-50 mg/mL in water) and drying for ~20 min. These thin films were used for room temperature electrical/thermoelectric measurements, scanning electron microscopy, X-ray diffraction, and X-ray photoelectron spectroscopy studies. Before use, glass substrates were washed. Typical cleaning procedure involved 15 min sonication in a bath of water, then acetone, then isopropanol. Substrates were then dried using nitrogen and placed in a UV-ozone cleaner for 10 min to improve hydrophilicity of the substrate surface and promote high-quality film formation.

Characterization

Scanning electron microscopy (SEM), transmission electron microscopy (TEM), X-ray diffraction (XRD), and x-ray photoelectron spectroscopy (XPS) were used to probe the structure and composition of the PEDOT:PSS-Te NW and PEDOT:PSS-Te(Cu_x) heterostructures and confirm that all materials are consistent with our previous reports. Inductively coupled plasma optical emission spectroscopy (ICP-OES) was used to measure the copper and tellurium composition of each sample.

A Zeiss Gemini Ultra-55 Analytical Field Emission Scanning Electron Microscope was used to perform SEM imaging (beam energy of 5 kV, In-Lens detector). TEM images were

recorded using a JEOL 2100-F Field-Emission Analytical TEM at 200 kV respectively. TEM samples were prepared by placing a TEM grid (400-mesh Cu or Ni on holey carbon – Ted Pella 01824) on a filter paper taped flat to a large aluminum block at 140°C. A single drop of dilute nanowire solution was deposited onto the grid and the water was allowed to evaporate for 5 min. XRD was performed using a Bruker AXS D8 Discover GADDS XRD micro-diffractometer. Wide-angle spectra were collected using a Co-Kα source at 0.179 nm wavelength. XPS spectra were collected using a Thermo Scientific K-Alpha XPS System with a monochromated Al Kα source. Pass energy of 100 eV was used for survey scans and 20 eV for high resolution scans. Thin film surfaces were cleaned using an argon cluster gun (6000 eV, 150 atoms per cluster) for 60 seconds before all XPS measurements.

Elemental analysis of each sample was performed using a Varian ICP-OES 720 Series. After purification of each NW sample, a small amount (1-5 mg) was digested using concentrated nitric acid for 72 hours. Samples were then diluted to 2 wt% nitric acid and run, along with 6 standards each for Cu and Te. For compositional quantification, multiple readings were taken at each of three different characteristic emission wavelengths and averaged. This allows for high accuracy determination of relative amounts of Cu and Te in each sample.

Room temperature electrical conductivity and Seebeck coefficient measurements

Room temperature electrical and thermoelectric measurements were performed using a home-built probe station. Thermal evaporation was used to pattern gold contacts (100nm) onto each of the four corners of the dropcast films using a shadow mask. Ohmic contacts were confirmed before all room temperature measurements. Sheet resistance of each film was measured using Keithley 2400 Sourcemeters in 4-wire van-der-Pauw configuration. For measurement of room temperature Seebeck coefficient (thermopower), two Peltier devices (Ferrotec) were placed ~4mm apart. A single current was passed through them in opposite polarities, which causes one device to heat and the other to cool by approximately the same amount). Thus, a sample placed across the two Peltiers experiences a temperature difference ΔT proportional to the current passed. Thermal paste (Wakefield Thermal S3 Solutions) was used to ensure good thermal contact, and the resulting open circuit voltage was measured using an Agilent 34401 multimeter. The temperature gradient was measured using two T-type thermocouples mounted in micromanipulators. Typically, five different temperature gradients were established (allowed to

equilibrate for 200 sec between temperature changes), with 10 voltage measurements taken and averaged at each ΔT. All samples exhibited linear variation of open circuit voltage with ΔT; this trend was used to extract Seebeck coefficient values. Data for both electrical conductivity and Seebeck coefficient were acquired using homemade Labview programs. Profilometry (Veeco Dektak 150 profilometer) was used to measure film thickness by scratching the film in several places and measuring the average step height. Electrical conductivity was extracted from the sheet resistance and thickness measurements.

Temperature dependent electrical conductivity and Seebeck measurements

The homemade thermoelectric measurement setup is used for electrical and thermoelectric characterization. Film was drop casted on quartz substrate (7x7 mm) pre-patterned with heater and measuring electrodes. The thickness of the film was measured using a KLA-Tencor profilometer. The electrical connections between the device and chip holder were made using a West Bond wire-bonder. The device was mounted on a 24-pin chip carrier to perform temperature dependent electrical and thermoelectric measurements. A Lakeshore 335 controller was used to control the base temperature of the device with an accuracy of 30 mK (at 300 K). A silicon diode sensor was placed on top of the chip carrier in close proximity with the chip to read the actual temperature of the device. Current was passed to the heater using a Keithley 2450 source meter to create a temperature gradient across the sample length. The resistance of both thermometers changes with change in temperature, i.e. change in heater current. Two SRS 830 lock-in amplifiers were used to read the resistance change of thermometers in a four probe configuration. Frequencies of the voltage output from the lock-in amplifiers were chosen as 781 Hz and 1107 Hz, respectively, with a 100 k high-precision resistor (to function as a current source) placed in series with each thermometer. The open circuit voltage (VOC) was read using a Keithley 2182 nano-voltmeter—this can be with an accuracy of nano volts.

Theoretical Methods

Te and $Cu_{1.75}Te$ nanowire surfaces are constructed according to the TEM and HR-TEM images in this study and in the literature.[5,10–12]

Degenerate and non-degenerate region:

The degenerate and non-degenerate lines originate from the Kang-Snyder charge transport model (CT) itself by applying degenerate (η>>1) and non-degenerate (η<<1) conditions. From CT model, conductivity and Seebeck can be written as:

$$\sigma = \sigma_{E_0}(T) \times sF_{s-1}(\eta) \qquad (2)$$

$$S = \frac{k_B}{e}\left[\frac{(s+1)F_s(\eta)}{sF_{s-1}(\eta)} - \eta\right] \qquad (3)$$

In degenerate region when (η>>1), conductivity and Seebeck can be written as :

$$\sigma = \sigma_{E_0}(T) \times \eta^s \qquad (4)$$

$$S = \frac{k_B}{e}\frac{\pi^2}{3} s \left(\frac{\sigma}{\sigma_{E_0}}\right)^{-\frac{1}{s}} \qquad (5)$$

In non-degenerate region when (η<<1), conductivity and Seebeck can be written as :

$$\sigma = \sigma_{E_0}(T) \times s\,\Gamma(s)\; exp(\eta) \qquad (6)$$

$$S = \frac{k_B}{e}\left[s + 1 - \ln(\frac{\sigma}{\sigma_{E_0} s\,\Gamma(s)})\right] \qquad (7)$$

Supplementary References

1. Shi, H., Liu, C., Jiang, Q. & Xu, J. Effective Approaches to Improve the Electrical Conductivity of PEDOT:PSS: A Review. *Adv. Electron. Mater.* **1,** 1500017 (2015).
2. Zhu, Z., Liu, C., Jiang, F., Xu, J. & Liu, E. Effective treatment methods on PEDOT:PSS to enhance its thermoelectric performance. *Synth. Met.* **225,** 31–40 (2017).
3. Zotti, G. *et al.* Electrochemical and XPS Studies toward the Role of Monomeric and Polymeric Sulfonate Counterions in the Synthesis, Composition, and Properties of Poly(3,4-ethylenedioxythiophene). *Macromolecules* **36,** 3337–3344 (2003).
4. Wang, Y. *et al.* PEDOT:PSS Modification by blending graphene oxide to improve the efficiency of organic solar cells. *Polym. Compos.* **39,** 3066–3072 (2017).
5. Zhou, C. *et al.* Nanowires as Building Blocks to Fabricate Flexible Thermoelectric Fabric: The Case of Copper Telluride Nanowires. *ACS Appl. Mater. Interfaces* **7,** 21015–21020 (2015).
6. Kang, S. D. & Snyder, G. J. Charge-transport model for conducting polymers. *Nat. Mater.* **16,** 252–257 (2017).
7. Park, Y. W. Structure and morphology: relation to thermopower properties of conductive polymers. *Synth. Met.* **45,** 173–182 (1991).
8. See, K. C. *et al.* Water-Processable Polymer−Nanocrystal Hybrids for Thermoelectrics. *Nano Lett.* **10,** 4664–4667 (2010).
9. Zaia, E. W. *et al.* Carrier Scattering at Alloy Nanointerfaces Enhances Power Factor in PEDOT:PSS Hybrid Thermoelectrics. *Nano Lett.* **16,** 3352–3359 (2016).
10. Park, H. *et al.* Aqueous chemical synthesis of tellurium nanowires using a polymeric template for thermoelectric materials. *CrystEngComm* **17,** 1092–1097 (2015).

11. Safdar, M. *et al.* Site-specific nucleation and controlled growth of a vertical tellurium nanowire array for high performance field emitters. *Nanotechnology* **24,** 185705 (2013).

12. Li, Z. *et al.* Controlled synthesis of tellurium nanowires and nanotubes via a facile, efficient, and relatively green solution phase method. *J. Mater. Chem. A* **1,** 15046–15052 (2013).
