# Supplementary material for: Polymer morphology and interfacial charge transfer dominate over energy-dependent scattering in organic-inorganic thermoelectrics
Source: Nat Commun. 2018 Dec 17;9:5347. doi: 10.1038/s41467-018-07435-z (PMC6297356; doi:10.1038/s41467-018-07435-z)
Supplement: Supplementary file 3 — Description of Additional Supplementary Files [file 41467_2018_7435_MOESM3_ESM.pdf]

Description of Additional Supplementary Files

File Name: Supplementary Movie 1
Description: Molecular Dynamics simulation of PEDOT18 (5 chains) and PSS36 (5 chains) on a Te surface depicts PEDOT templating at the organic-inorganic interface. This effect is only observed in the first few layers of PEDOT molecules at the Te surface.

File Name: Supplementary Movie 2
Description: Molecular Dynamics simulation of PEDOT18 (5 chains) and PSS36 (5 chains) on a Cu1.75Te surface also depicts strong templating of PEDOT at the organic-inorganic interface.

File Name: Supplementary Movie 3
Description: Molecular Dynamics simulation of pristine PEDOT18 (20 chains) on Te surface. Multi-layer templating of PEDOT is observed on the inorganic surface.

File Name: Supplementary Movie 4
Description: Molecular Dynamics simulation of pristine PEDOT18 (20 chains) on Cu1.75Te surface. A similar effect is observed as in the Te case.

File Name: Supplementary Movie 5
Description: Molecular Dynamics simulation of pristine PSS36 (20 chains) on Cu1.75Te surface. PSS is not observed to interact strongly with or form local morphologies at the inorganic surface.

File Name: Supplementary Movie 6
Description: Molecular Dynamics simulation shows that, two distinct PEDOT18 aggregates on a Te surface tend to form a single percolating PEDOT domain.

File Name: Supplementary Movie 7
Description: Molecular Dynamics simulation indicates that a "kink" in the inorganic nanostructure disrupts the ability of two local PEDOT regimes to form a single percolating network.

File Name: Supplementary Movie 8
Description: Upon simulated annealing, PEDOT18 chains are observed to self-assemble on Te surface.

File Name: Supplementary Movie 9
Description: Upon simulated annealing, no self-assembly of PEDOT18 chains is observed on Cu1.75Te surface. This phenomenon is attributed to stronger PEDOT- Cu1.75Te interactions.
